# Detection of overdose and underdose prescriptions—An unsupervised machine learning approach

**DOI:** 10.1371/journal.pone.0260315

**Published:** 2021-11-19

**Authors:** Kenichiro Nagata, Toshikazu Tsuji, Kimitaka Suetsugu, Kayoko Muraoka, Hiroyuki Watanabe, Akiko Kanaya, Nobuaki Egashira, Ichiro Ieiri

**Affiliations:** 1 Department of Pharmacy, Kyushu University Hospital, Fukuoka, Japan; 2 Department of Pharmacy, Fukuoka Tokushukai Hospital, Fukuoka, Japan; Frederick National Laboratory for Cancer Research, UNITED STATES

## Abstract

Overdose prescription errors sometimes cause serious life-threatening adverse drug events, while underdose errors lead to diminished therapeutic effects. Therefore, it is important to detect and prevent these errors. In the present study, we used the one-class support vector machine (OCSVM), one of the most common unsupervised machine learning algorithms for anomaly detection, to identify overdose and underdose prescriptions. We extracted prescription data from electronic health records in Kyushu University Hospital between January 1, 2014 and December 31, 2019. We constructed an OCSVM model for each of the 21 candidate drugs using three features: age, weight, and dose. Clinical overdose and underdose prescriptions, which were identified and rectified by pharmacists before administration, were collected. Synthetic overdose and underdose prescriptions were created using the maximum and minimum doses, defined by drug labels or the UpToDate database. We applied these prescription data to the OCSVM model and evaluated its detection performance. We also performed comparative analysis with other unsupervised outlier detection algorithms (local outlier factor, isolation forest, and robust covariance). Twenty-seven out of 31 clinical overdose and underdose prescriptions (87.1%) were detected as abnormal by the model. The constructed OCSVM models showed high performance for detecting synthetic overdose prescriptions (precision 0.986, recall 0.964, and F-measure 0.973) and synthetic underdose prescriptions (precision 0.980, recall 0.794, and F-measure 0.839). In comparative analysis, OCSVM showed the best performance. Our models detected the majority of clinical overdose and underdose prescriptions and demonstrated high performance in synthetic data analysis. OCSVM models, constructed using features such as age, weight, and dose, are useful for detecting overdose and underdose prescriptions.

## Introduction

Prescription errors that occur in hospitals cause adverse drug events (ADEs), that may occasionally result in death [1]. In a recent systematic review, the frequency of prescription errors was at least 2%, while that of preventable ADEs was estimated to be 0.4% [2]. The World Health Organization has announced its global patient safety challenge, which aims to reduce medication-related harm by 50% within five years by improving unsafe practices and reducing medication errors [3]. Prescription errors related to drug overdose may result in serious life-threatening ADEs, while those related to the underdosing of drugs may lead to diminished therapeutic effects. Thus, it is particularly important to detect and prevent these errors before the administration of drugs [4, 5]. Previous studies suggested that the implementation of Electronic Health Records (EHRs) with Clinical Decision Support (CDS) systems is useful for detecting and preventing prescription errors, including overdoses and underdoses [6–13]. However, current CDS systems have two main limitations. The first issue is that most of these systems are rule-based and can thus only detect prescription errors according to pre-programmed rules. Moreover, in the case of insufficient information from reliable sources (e.g., a lack of pediatric dosage information in a drug label), difficulties are associated with making rules. The second issue is that current CDS systems may raise too many false-positive alerts, which result in medical staff habitually overriding them [14, 15]. This is called “alert fatigue.” Therefore, the development of more precise CDS systems is urgently needed [16–19]. To overcome these limitations, a non-rule-based novel approach is required.

In clinical practice, the majority of prescriptions are generally within the appropriate dose range, and overdose and underdose prescriptions are extremely rare [5]. Thus, the detection of abnormal prescriptions involves the identification of a small amount of abnormal data among mostly normal data. This issue has been examined as unsupervised anomaly detection in the field of machine learning [20].

## Related works

To the best of our knowledge, MedAware (Raanana, Israel) is the first commercial system for preventing prescription errors by utilizing machine learning techniques [21]. This system enables the generation of automatic alerts by analyzing EHRs and detects overdose and underdose prescriptions with low false-positive rates [22–24]. Segal et al. introduced a machine learning based CDS system (MedAware) in clinical practice and evaluated its usefulness [23]. The system analyzed 78 017 prescriptions, generated 282 alerts (0.4%), and resulted in discontinuation or change in 135 prescriptions. However, this report does not provide information about the machine learning process, probably due to commercial reasons.

Santos et al. applied a graph centrality approach, known as density-distance-centrality (DDC), for outlier detection to identify overdose and underdose prescriptions [25]. They showed that DDC achieved better results than typical unsupervised machine learning techniques [25]. However, they only used two features, “dose” and “daily frequency,” and did not consider “age” and “weight,” which are critical factors for clinical dosage adjustments [25]. Recently, they developed a SaaS (software as a service) called NoHarm.ai which enables screening for non-standard prescriptions by analyzing hospital data [26].

Corny et al. proposed a hybrid CDS system based on a rule-based technique and supervised machine learning approach [27]. They combined patient-related data (e.g., age, weight, sex) and rule-based alerts (e.g., dosage, frequency, route) for each prescription with labeling (binary: 1 = a pharmaceutical intervention; 0 = no pharmaceutical intervention) and used it as training data. Using LightGBM, a gradient-boosting framework based on decision tree algorithms, predicted scores at the patient level were calculated. Their hybrid CDS system showed higher performance than the classic CDS system (F-measure 0.74 vs. 0.61). However, in the clinical applicability of CDS systems, it is challenging to obtain precisely labeled data for a huge number of prescriptions. In addition, because their method starts from the rule-based technique, it requires pre-programmed rules. If it is difficult to make rules due to insufficient information (e.g., a lack of pediatric dosage information in a drug label), their system will not work. Unsupervised machine learning algorithms can be used to solve these problems.

As far as we know, there have been no further attempts to detect prescription errors of overdoses and underdoses using machine learning. In order to establish a method for detecting drug overdose and underdose using machine learning, open discussions with detailed explanations and analysis codes are necessary.

The purpose of this study was to detect extreme overdose and underdose prescriptions that occur very rarely in clinical practice using unsupervised machine learning algorithms. We constructed models for each candidate drug using three features: age, weight, and dose, and evaluated their usefulness for detecting overdose and underdose prescriptions.

## Methods

This study was approved by the Ethics Committee of the Kyushu University Hospital (approval number 2020–187). All data were fully anonymized before access and the ethics committee waived the requirement for informed consent.

### Investigation of clinical overdose and underdose prescriptions

Clinical overdose and underdose prescriptions, which were identified and rectified by pharmacists before dispensing in our hospital between January 1 and December 31, 2019, were collected. Thirty-one clinical overdose and underdose prescriptions (consisting of 21 drugs) that met the following conditions were analyzed:

- oral drugs
- more than 1000 in-hospital prescriptions between January 1, 2014 and December 31, 2019 (to ensure sufficient training data for the construction of the OCSVM model)
- based on drug labels or the UpToDate database [28], the maximum dose and minimum dose could be defined according to age, weight, or both (to create synthetic overdose and underdose prescriptions to evaluate OCSVM model performance)

### Data preprocessing

We extracted prescription data and weight data from EHRs between January 1, 2014 and December 31, 2019. In terms of weight data, patients less than 0 kg or more than 300 kg were excluded because they were considered to be input errors. In prescription data, the dose was converted to the value corresponding to the amount of the active ingredient. Each set of prescription data was linked to the closest weight data, and data that met the following conditions were included in the analysis:

- prescriptions of 21 drugs (identified in the previous section)
- in-hospital prescriptions
- ordered in daily dose (prescription data entered by “single dose taken when needed” and “total dose” were excluded because the actual dose taken by patient was unknown)
- weight data existed within 90 days before or after prescription

### OCSVM methodology

The OCSVM methodology was initially proposed by Schölkopf et al. [29] OCSVM requires the majority of training data to be normal, fits a hyperplane to include the majority of training data, and detects abnormal data as deviations from the decision boundary. First, the method maps the training data into the feature space corresponding to a simple kernel

$$k\left(x_{i},x_{j}\right)=\left(\Phi \left(x_{i}\right)\cdot \Phi \left(x_{j}\right)\right)$$ (1)

such as the radial basis function (RBF) kernel

$$k\left(x_{i},x_{j}\right)=exp\left(-\gamma {\left\|x_{i}-x_{j}\right\|}^{2}\right),\;\gamma >0$$ (2)

where γ represents a kernel coefficient.

To separate the data from the origin, the following dual problem was solved

$$\underset{\alpha }{\text{min}}\frac{1}{2}\underset{ij}{\sum }\alpha_{i}\alpha_{j}k\left(x_{i},x_{j}\right)$$ (3)

subject to

$$0\leq \alpha_{i}\leq \frac{1}{\nu l},\;\underset{i}{\sum }\alpha_{i}=1.$$ (4)

Here, *α*_*i*_ is a Lagrange multiplier, *ν* defines the maximum fraction of outliers in training data, *l* is the number of points in the training dataset. The resulting decision function can be expressed as

$$f\left(x\right)=\underset{i}{\sum }\alpha_{i}k\left(x_{i},x\right)-\rho$$ (5)

where the offset ρ can be obtained as

$$\rho =\underset{j}{\sum }\alpha_{j}k\left(x_{j},x_{i}\right).$$ (6)

In OCSVM, we employed the implementation available in scikit-learn 0.22.1 [30]. We used the RBF as a kernel trick. The performance of OCSVM with the RBF kernel is strongly influenced by two hyperparameters: *ν* and γ. The incidence of clinical overdose and underdose prescriptions for each drug was between 0.01% and 0.44% (S1 Table). Therefore, we set the *ν* value to 1% (0.01) in the present study. The hyperparameter γ affects the influence area of the support vectors on the classification. In general, increasing the value of γ implies adjusting the frontier closer to the training data and improving recall. However, a marked increase in γ causes overfitting of the model to the training data and deteriorates precision. The hyperparameter γ was set to “scale,” which is the default setting in scikit-learn 0.22.1, and calculated as follows:

$$\gamma =\frac{1}{\text{f}\cdot \text{v}}$$ (7)

where f is the number of features and v represents the variance in the dose of training data.

### Experiment 1: Evaluation of OCSVM model performance for clinical overdose and underdose prescriptions

Age, weight, and daily dose were extracted as features from the prescription data between January 1, 2014 and December 31, 2019. We used these prescription data as training data and constructed an OCSVM model for each drug (total of 21 models). In each clinical overdose and underdose prescription (total of 31 prescriptions), age, weight, and daily dose were standardized by removing the mean and scaling to unit variance based on the training data using the StandardScaler module in scikit-learn and we applied it to the OCSVM model. The OCSVM model returned the signed distance to the separating hyperplane and predicted whether each prescription was normal (positive value, inside the decision boundary) or abnormal (negative value, outside the decision boundary).

### Experiment 2: Evaluation of OCSVM model performance for synthetic overdose and underdose prescriptions

To ensure sufficient data for the evaluation of OCSVM model performance, we created synthetic overdose and underdose prescriptions and conducted a five-fold cross-validation analysis for each drug. The maximum dose and minimum dose according to age, weight, or both were defined based primarily on drug labels and secondarily on UpToDate (when there was insufficient information in the drug labels). The entire dataset (prescription data between January 1, 2014 and December 31, 2019) was randomly divided into five folds, and four folds of the dataset were used as training data. From one-fold of the dataset, we randomly selected 50% that were within the maximum and minimum doses and used them as normal prescriptions. Regarding the other 50% of the dataset, we artificially changed the daily dose to 2 times the maximum dose for synthetic overdose prescriptions and to 0.1 times the minimum dose for synthetic underdose prescriptions. Data (age, weight, and daily dose) on normal prescriptions and synthetic overdose and underdose prescriptions were standardized based on training data and applied to the OCSVM model of the corresponding drug. To evaluate OCSVM model performance, we used the following metrics:

- True positives (TP): the number of synthetic overdose or underdose prescriptions correctly predicted as abnormal
- False positives (FP): the number of normal prescriptions incorrectly predicted as abnormal
- True negatives (TN): the number of normal prescriptions correctly predicted as normal
- False negatives (FN): the number of synthetic overdose or underdose prescriptions incorrectly predicted as normal

$$\text{Precision}=\frac{\text{TP}}{\left(\text{TP}+\text{FP}\right)}$$ (8)

$$\text{Recall}=\frac{\text{TP}}{\left(\text{TP}+\text{FN}\right)}$$ (9)

$$\text{F-measure}=\frac{2\cdot \text{precision}\cdot \text{recall}}{\left(\text{precision}+\text{recall}\right)}$$ (10)

Random selection was repeated 10 times, and the average value was calculated to obtain robust results. The overall performance of OCSVM models was evaluated based on the average of the metrics for 21 drugs. We changed the γ value logarithmically from 2^−6^ to 2^6^ and examined its influence on the OCSVM model performance.

To plot graphs, we used GraphPad Prism ver.8.4.1 for Windows (GraphPad Software, San Diego, CA, USA) for a line plot and Matplotlib 3.1.1, scikit-image 0.16.2, and MATLAB version 9.10.0.1602886 (The MathWorks Inc, Natick, MA, USA) for a three-dimensional plot [31, 32].

### Experiment 3: Comparative analysis with unsupervised outlier detection algorithms

To compare model performance between OCSVM and other unsupervised outlier detection algorithms, we used the following methods.

- Local outlier factor (LOF): It measures the local density deviation of a given data point with respect to its neighbors [33]. The LOF score of an observation is equal to the ratio of the average local density of k-nearest neighbors and its own local density. It depends on hyperparameters: k (number of neighbors) and contamination (proportion of outliers in the data set).
- Isolation forest (ISO): It isolates observations by randomly selecting a feature and then randomly selecting a split value of the selected feature [34]. The number of splitting required to isolate a sample is equal to the path length from the root to the terminating node. This path length is a measure of normality and decision function. It depends on hyperparameters: estimators (number of base estimators in the ensemble) and contamination.
- Robust covariance (RC): Assuming gaussian distribution, it estimates the inlier location and covariance without being influenced by outliers [35]. The Mahalanobis distances obtained from this estimate are used to measure deviation. It depends on the hyperparameter of contamination.

We used synthetic overdose and underdose prescriptions created in Experiment 2 and conducted a five-fold cross-validation analysis using OCSVM, LOF, ISO and RC. We changed the value of hyperparameters as shown in Table 1, and the best F-measure was compared between algorithms.

**Table 1:** Hyperparameters evaluated to find the best F-measure.

Algorithm	Hyperparameter	Value
OCSVM	γ	2^−6^, 2^−5^, 2^−4^, 2^−3^, 2^−2^, 2^−1^, 1, 2, 4, 8
	*ν*	2^−10^, 2^−9^, 2^−8^, 2^−7^, 2^−6^, 2^−5^, 2^−4^
LOF	k	10, 20, 30, 40, 50, 60, 70, 80, 90, 100
	contamination	2^−10^, 2^−9^, 2^−8^, 2^−7^, 2^−6^, 2^−5^, 2^−4^
ISO	estimators	10, 20, 30, 40, 50, 60, 70, 80, 90, 100
	contamination	2^−10^, 2^−9^, 2^−8^, 2^−7^, 2^−6^, 2^−5^, 2^−4^
RC	contamination	2^−10^, 2^−9^, 2^−8^, 2^−7^, 2^−6^, 2^−5^, 2^−4^

## Results

### Experiment 1: Evaluation of OCSVM model performance for clinical overdose and underdose prescriptions

Details regarding the clinical overdose and underdose prescriptions that were prevented by pharmacists in 2019 are shown in Table 2. Thirty-one (20 overdose and 11 underdose) prescriptions of 21 drugs were analyzed. To assess the degree of the deviation of clinical overdose and underdose prescriptions, the ratio to the maximum or minimum dose was calculated (Table 2). Regarding clinical overdose prescriptions, the median (range) of the ratio to the maximum dose was 1.88 (1.25–14.49). In the case of clinical underdose prescriptions, the median (range) of the ratio to the minimum dose was 0.13 (0.001–0.65). Twenty-seven out of 31 clinical overdose and underdose prescriptions (87.1%) were detected as abnormal by the OCSVM models.

**Table 2:** Details of clinical overdose and underdose prescriptions and detection results by OCSVM models.

Drug name (strength)	Age	Weight (kg)	Dose (/day)	O/U^a^	Ratio to max/min^b^	OCSVM^c^
Acetaminophen fine granule (500 mg/g)	64	49.6	3 mg	U	0.02	+
	68	51.1	3 mg	U	0.02	+
Ambroxol hydrochloride dry syrup (15 mg/g)	0	3.6	1.05 mg	U	0.65	-
	0	2.6	8 mg	O	1.71	-
	1	10.8	90 mg	O	4.63	+
Amlodipine besylate tablet (5 mg/tablet)	71	74.9	20 mg	O	2.00	+
Aprepitant capsule (80 mg/capsule)	59	51.6	160 mg	O	1.28	+
Aspirin tablet (100 mg/tablet)	81	43.0	10 000 mg	O	2.33	+
Calcium carbonate tablet (500 mg/tablet)	5	8.4	0.75 mg	U	0.001	+
Carvedilol tablet (10 mg/tablet)	13	31.4	55 mg	O	1.38	+
	70	55.8	200 mg	O	5.00	+
Celecoxib tablet (200 mg/tablet)	54	59.1	800 mg	O	1.33	-
Codeine phosphate powder (10 mg/g)	51	52.7	6 mg	U	0.20	+
	69	61.2	6 mg	U	0.20	+
	85	49.9	4 mg	U	0.13	+
Furosemide fine granule (40 mg/g)	0	0.46	40 mg	O	14.49	+
	13	30.0	0.25 mg	U	0.02	-
Lactulose syrup (0.65 g/ml)	82	42.6	1.95 g	U	0.20	+
Levothyroxine sodium hydrate tablet (25 μg/tablet)	1	10.7	250 μg	O	3.89	+
Nicorandil tablet (5 mg/tablet)	66	38.7	75 mg	O	2.50	+
Nifedipine sustained release tablet (10 mg/tablet)	47	69.5	140 mg	O	1.75	+
Omeprazole tablet (10 mg/tablet)	4	10.9	20 mg	O	2.00	+
Phenobarbital powder (100 mg/g)	66	53.8	500 mg	O	1.25	+
Rabeprazole sodium tablet (10 mg/tablet)	20	48.5	70 mg	O	1.75	+
	58	74.8	70 mg	O	1.75	+
	68	48.4	100 mg	O	2.50	+
Rivaroxaban tablet (15 mg/tablet)	57	54.0	45 mg	O	1.50	+
Spironolactone fine granule (100 mg/g)	13	30.0	0.05 mg	U	0.002	+
	74	47.5	500 mg	O	2.50	+
Trimethoprim sulfamethoxazole granule^d^ (80 mg/g)	0	3.5	1.6 mg	U	0.23	+
Ursodeoxycholic acid granule (50 mg/g)	2	12.0	540 mg	O	1.50	+

^a^ O, overdose; U, underdose.

^b^ max, maximum dose; min, minimum dose defined by drug labels or UpToDate. The ratio to the maximum dose is shown for overdose. The ratio to the minimum dose is shown for underdose.

^c^ “+” indicates detected, “-” indicates not detected as abnormal prescriptions, respectively.

^d^ Dose is the value equivalent to trimethoprim.

### Experiment 2: Evaluation of OCSVM model performance for synthetic overdose and underdose prescriptions

The performance of the OCSVM model for synthetic overdose and underdose prescriptions for each drug is shown in Table 3.

**Table 3:** OCSVM model performance for detecting synthetic overdose and underdose prescriptions for each drug.

Drug name (strength)	Data (n)	Synthetic overdose prescriptions			Synthetic underdose prescriptions		
		Precision	Recall	F-measure	Precision	Recall	F-measure
Acetaminophen fine granule (500 mg/g)	5869	0.986	1.000	0.993	0.986	0.969	0.977
Ambroxol hydrochloride dry syrup (15 mg/g)	4067	0.991	0.949	0.969	0.971	0.288	0.443
Amlodipine besylate tablet (5 mg/tablet)	37 796	0.991	0.997	0.994	0.991	1.000	0.996
Aprepitant capsule (80 mg/capsule)	14 436	0.989	1.000	0.995	0.989	1.000	0.995
Aspirin tablet (100 mg/tablet)	38 736	0.991	1.000	0.995	0.991	1.000	0.995
Calcium carbonate tablet (500 mg/tablet)	5049	0.987	0.974	0.981	0.987	0.932	0.958
Carvedilol tablet (10 mg/tablet)	3603	0.983	1.000	0.991	0.983	1.000	0.991
Celecoxib tablet (200 mg/tablet)	13 205	0.989	0.992	0.991	0.989	1.000	0.994
Codeine phosphate powder (10 mg/g)	4104	0.985	1.000	0.993	0.985	0.957	0.970
Furosemide fine granule (40 mg/g)	3366	0.988	0.930	0.958	0.958	0.251	0.397
Lactulose syrup (0.65 g/ml)	3243	0.987	1.000	0.993	0.986	0.940	0.962
Levothyroxine sodium hydrate tablet (25 μg/tablet)	15 467	0.991	0.996	0.993	0.990	0.924	0.956
Nicorandil tablet (5 mg/tablet)	5669	0.986	1.000	0.993	0.986	1.000	0.993
Nifedipine sustained release tablet (10 mg/tablet)	2088	0.976	1.000	0.988	0.976	1.000	0.988
Omeprazole tablet (10 mg/tablet)	5965	0.986	0.994	0.990	0.986	1.000	0.993
Phenobarbital powder (100 mg/g)	2399	0.978	0.703	0.817	0.969	0.479	0.638
Rabeprazole sodium tablet (10 mg/tablet)	54 423	0.989	1.000	0.995	0.989	1.000	0.995
Rivaroxaban tablet (15 mg/tablet)	2022	0.980	1.000	0.990	0.980	1.000	0.990
Spironolactone fine granule (100 mg/g)	11 379	0.987	0.879	0.930	0.968	0.346	0.510
Trimethoprim sulfamethoxazole granule^a^ (80 mg/g)	5683	0.990	1.000	0.995	0.944	0.178	0.298
Ursodeoxycholic acid granule (50 mg/g)	7669	0.986	0.825	0.899	0.973	0.418	0.584

Data represent the average values of ten repeats of five-fold cross-validation.

^a^ Dose is the value equivalent to trimethoprim.

**Fig 1:**
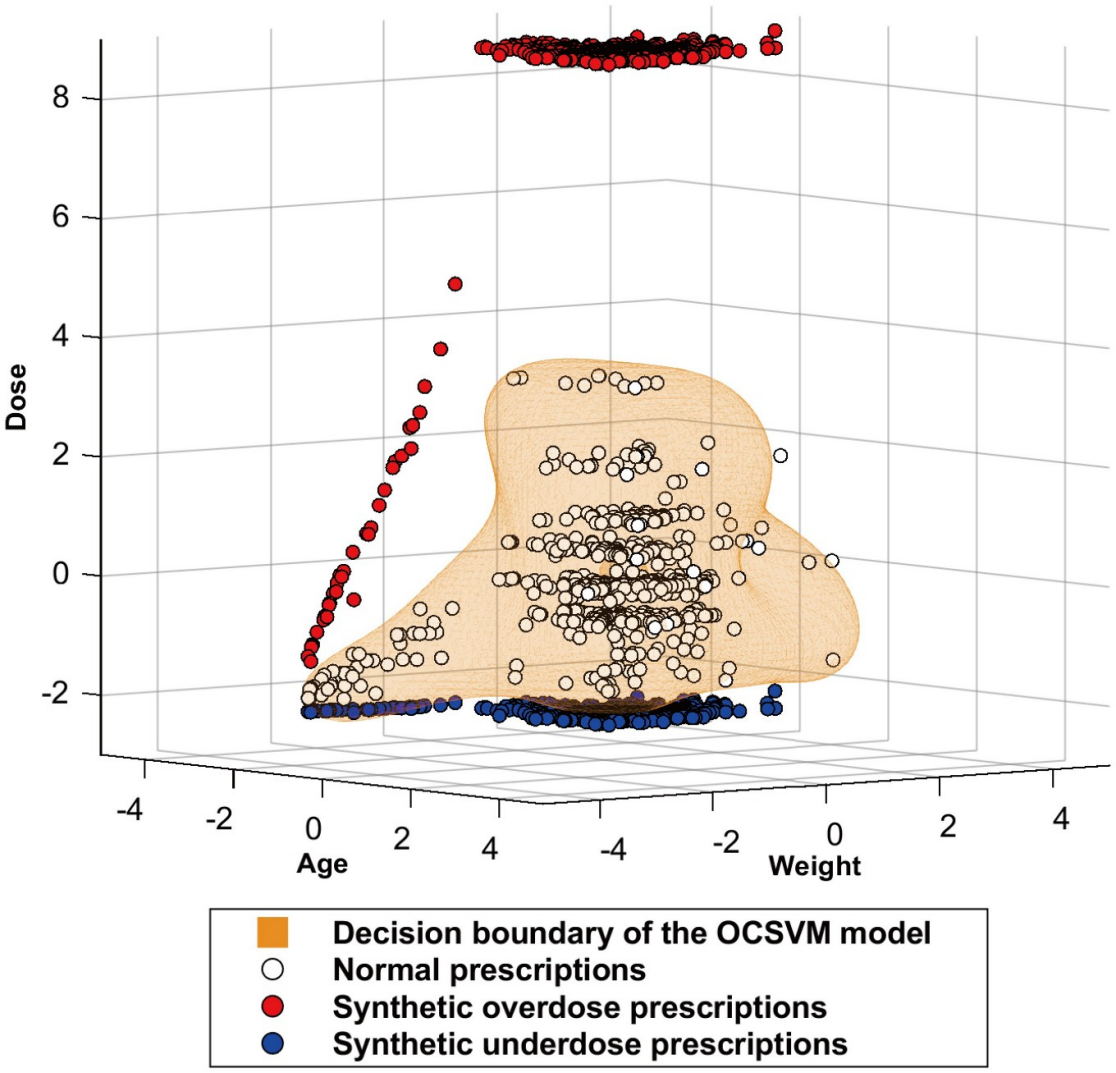
Decision boundary of the OCSVM model and individual data for acetaminophen fine granules. Data are represented as standardized values.

We plotted each prescription data and the decision boundary of the OCSVM model for acetaminophen fine granules in Fig 1. The results showed that the majority of normal prescriptions were inside the decision boundary, and all synthetic overdose prescriptions and most synthetic underdose prescriptions were outside the decision boundary.

The overall performance of the OCSVM models for synthetic overdose and underdose prescriptions is shown in Table 4.

**Table 4:** Overall performance of OCSVM models for synthetic overdose and underdose prescriptions.

	Precision	Recall	F-measure
Synthetic overdose prescriptions	0.986	0.964	0.973
Synthetic underdose prescriptions	0.980	0.794	0.839

Data represent the average values of 21 drugs.

**Fig 2:**
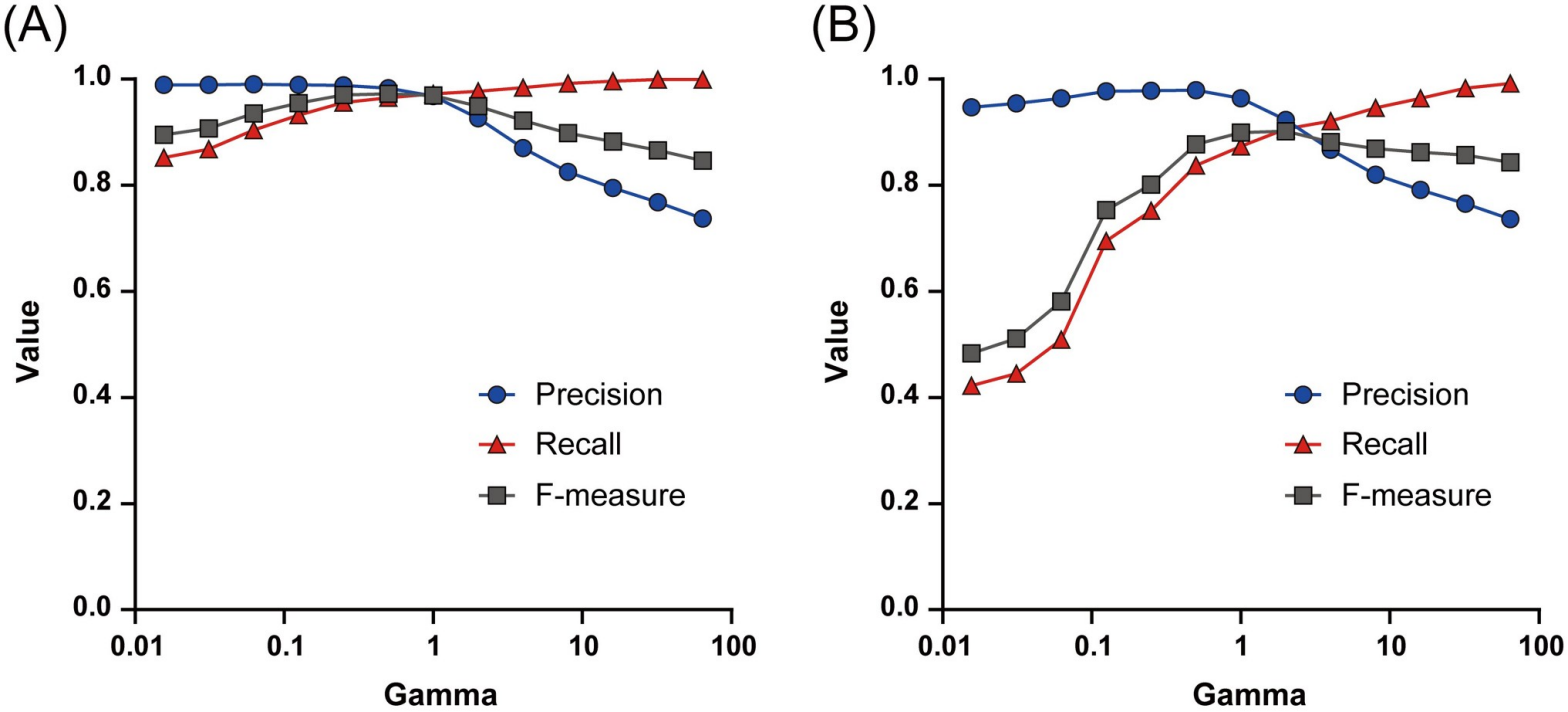
Influence of hyperparameter γ on overall performance of OCSVM models. (A) Analysis for synthetic overdose prescriptions. (B) Analysis for synthetic underdose prescriptions.

The influences of the hyperparameter γ on the OCSVM model performance for synthetic overdose and underdose prescriptions are shown in Fig 2A and 2B. Precision and recall were inversely related, that is, the smaller the γ value, the higher the precision; and the larger the γ value, the higher the recall.

### Experiment 3: Comparative analysis with unsupervised outlier detection algorithms

The optimized hyperparameters and model performance of OCSVM, LOF, ISO and RC for synthetic overdose and underdose prescriptions are shown in Table 5.

**Table 5:** Comparative analysis with unsupervised anomaly detection algorithms.

	Algorithm	Optimized hyperparameters	Precision	Recall	F-measure
Synthetic overdose prescriptions	OCSVM	γ = 2^−1^, *ν* = 2^−6^	0.980	0.969	0.973
	LOF	k = 40, contamination = 2^−5^	0.968	0.932	0.942
	ISO	estimators = 30, contamination = 2^−4^	0.897	0.746	0.784
	RC	contamination = 2^−4^	0.874	0.763	0.781
Synthetic underdose prescriptions	OCSVM	γ = 2, *ν* = 2^−5^	0.934	0.919	0.918
	LOF	k = 60, contamination = 2^−4^	0.931	0.879	0.895
	ISO	estimators = 20, contamination = 2^−4^	0.785	0.404	0.498
	RC	contamination = 2^−4^	0.706	0.312	0.375

Data represent the average values of 21 drugs.

## Discussion

In our investigation of clinical overdose and underdose prescriptions, 12 out of 31 prescriptions were for children or infants, and 9 out of 21 drugs were in powder or liquid forms (Table 2). These results suggest that it is important to take “age” and “weight” into consideration when detecting overdose or underdose prescription errors that occur in clinical settings. In a previous study, in which the detection of overdose and underdose prescriptions was attempted using a graph centrality approach and typical unsupervised machine learning techniques, only “dose” and “daily frequency” were used as the features [25]. Although difficulties are generally associated with comparing the findings of the aforementioned study to the present results due to the use of different methods, their model showed lower performance (F-measure: 0.68) [25]. In the present study, we demonstrated for the first time that by using the three simple features of “age,” “weight,” and “dose,” OCSVM models detected the majority of clinical overdose and underdose prescriptions (Table 2). Furthermore, the model demonstrated high performance in the synthetic data analysis (Table 4).

Difficulties are associated with obtaining sufficient clinical overdose and underdose prescription data to evaluate OCSVM model performance. Therefore, we defined maximum and minimum doses based on drug labels or UpToDate information and created synthetic overdose and underdose prescriptions. The factors of synthetic data (maximum dose × 2 or minimum dose × 0.1) were set according to the ratio of the clinical overdose to the maximum dose (median [range]: 1.88 [1.25–14.49]) or that of the clinical underdose to the minimum dose (0.13 [0.001–0.65]), as shown in Table 2, and were considered to be clinically feasible and of reasonable value.

In the analysis of OCSVM model performance (Table 3), all drugs showed high precision (> 0.94), which suggests that the low false-positive rate in our model avoided “alert fatigue.” Regarding synthetic overdose prescriptions, recall was > 0.82 for 20 out of 21 drugs and 0.703 for phenobarbital powder (Table 3). Among synthetic underdose prescriptions, recall was > 0.92 for 15 out of 21 drugs, but < 0.48 for six drugs, including phenobarbital powder (Table 3). In our hospital, phenobarbital powder is often used in quantities outside the dose range described in the drug label or UpToDate, particularly when administered to infants and children (age: 0–5), with careful therapeutic drug monitoring being implemented before drug administration. In the prescription data for phenobarbital powder, 9.6% was above the maximum dose, while 3.9% was below the minimum dose (S2 Table), which may have resulted in low recall and F-measure values. These results indicate that because of its inherent nature, the machine learning approach may not have the capacity to detect prescriptions that are not rare, but that also require attention, such as the confirmation of blood concentrations. This issue may be resolved by adding drug blood concentrations to the features of the OCSVM model.

Regarding the overall performance of OCSVM models, excellent results were obtained for synthetic overdose prescriptions. However, the performance was slightly lower for synthetic underdose prescriptions (Table 4). Clinically, patients at a high risk of developing ADEs are sometimes administered drugs at lower doses than the minimum dose described in the drug label or UpToDate. Additionally, even if only one dose is prescribed for a drug that is administered multiple times daily (e.g., dosing only after dinner on the start date), our model recognized it as a daily dose. These factors may have limited the detection of synthetic underdose prescriptions.

γ-dependent changes in the metrics are shown in Fig 2. F-measure peaked when γ was 2^−1^ for synthetic overdose prescriptions and 2^1^ for synthetic underdose prescriptions. Therefore, setting γ approximately between 0.5 and 2.0 was considered to be appropriate. Moreover, adjustments of γ for each drug (high γ setting to prioritize recall for high-risk drugs, and low γ setting to prioritize precision for low-risk drugs) may enhance the utility in clinical settings.

In comparative analysis with unsupervised outlier detection algorithms, OCSVM showed the best F-measure for synthetic overdose and underdose prescriptions. Because LOF also showed high performance, OCSVM and LOF were considered as suitable algorithms for detecting overdose and underdose prescriptions.

This study considered age and weight, which are the main factors affecting dosage. Careful evaluation of several other factors related to the dose of individual drug, such as renal function (creatinine clearance), drug blood concentrations, and other laboratory parameters, may improve the model’s utility in further studies. To verify the results, we need to show that our model has high detection performance even for different data sets (e.g., prescription data obtained from other hospitals). The results of the present study may have implications for development of a CDS system based on our method in real clinical settings. In future studies, the efficacy of the system should be evaluated for its utility in alerting medical staff and subsequent benefits in treatment, in comparison with the current rule-based systems.

## Conclusions

In the present study, we revealed that OCSVM models, constructed using three features: age, weight, and dose, detected the majority of clinical overdose and underdose prescriptions. Moreover, the models demonstrated high performance in the synthetic data analysis. These results suggest that our model is a useful CDS system for detecting prescription errors related to overdoses and underdoses. Further prospective studies are needed to assess the performance of the OCSVM model in real-world settings.

## Supporting information

S1 Table Occurrence rates of clinical overdose and underdose prescriptions for each drug. (DOCX)

S2 Table Details of prescription data for phenobarbital powder. (DOCX)

S1 File Data and analysis code. (ZIP)
