# Supplementary material for: Detection of overdose and underdose prescriptions—An unsupervised machine learning approach
Source: PLoS One. 2021 Nov 19;16(11):e0260315. doi: 10.1371/journal.pone.0260315 (PMC8604308; doi:10.1371/journal.pone.0260315)
Supplement: S1 Table — (DOCX) [file pone.0260315.s001.docx]

**S1 Table**

| **Drug name (strength)** | **Clinical overdose and underdose prescriptions^a^**  **(n)** | **Total prescriptions^b^**  **(n)** | **Occurrence rate**  **(%)** |
| --- | --- | --- | --- |
| Acetaminophen Fine Granule (500 mg/g) | 2 | 1421 | 0.14 |
| Ambroxol Hydrochloride Dry Syrup (15 mg/g) | 3 | 1057 | 0.28 |
| Amlodipine Besylate Tablet (5 mg/Tablet) | 1 | 7609 | 0.01 |
| Aprepitant Capsule (80 mg/Capsule) | 1 | 2687 | 0.04 |
| Aspirin Tablet (100 mg/Tablet) | 1 | 6273 | 0.02 |
| Calcium Carbonate Tablet (500 mg/Tablet) | 1 | 885 | 0.11 |
| Carvedilol Tablet (10 mg/Tablet) | 2 | 1460 | 0.14 |
| Celecoxib Tablet (200 mg/Tablet) | 1 | 2020 | 0.05 |
| Codeine Phosphate Powder (10 mg/g) | 3 | 683 | 0.44 |
| Furosemide Fine Granule (40 mg/g) | 2 | 1537 | 0.13 |
| Lactulose Syrup (0.65 g/mL) | 1 | 848 | 0.12 |
| Levothyroxine Sodium Hydrate Tablet (25 μg/Tablet) | 1 | 2816 | 0.04 |
| Nicorandil Tablet (5 mg/Tablet) | 1 | 1250 | 0.08 |
| Nifedipine Sustained Release Tablet (10 mg/Tablet) | 1 | 429 | 0.23 |
| Omeprazole Tablet (10 mg/Tablet) | 1 | 717 | 0.14 |
| Phenobarbital Powder (100 mg/g) | 1 | 557 | 0.18 |
| Rabeprazole Sodium Tablet (10 mg/Tablet) | 3 | 6689 | 0.04 |
| Rivaroxaban Tablet (15 mg/Tablet) | 1 | 370 | 0.27 |
| Spironolactone Fine Granule (100 mg/g) | 2 | 1960 | 0.10 |
| Trimethoprim Sulfamethoxazole Granule^*3^ (80 mg/g) | 1 | 1625 | 0.06 |
| Ursodeoxycholic Acid Granule (50 mg/g) | 1 | 1659 | 0.06 |

^a^ The number of clinical overdose and underdose prescriptions prevented by pharmacists before administration in 2019.

^b^ The total number of prescriptions in 2019 analyzed in the present study.

^c^ Dose is the value equivalent to trimethoprim.
