# Supplementary material for: Detection of overdose and underdose prescriptions—An unsupervised machine learning approach
Source: PLoS One. 2021 Nov 19;16(11):e0260315. doi: 10.1371/journal.pone.0260315 (PMC8604308; doi:10.1371/journal.pone.0260315)
Supplement: S2 Table — (DOCX) [file pone.0260315.s002.docx]

**S2 Table**

| **Age** | **Dose > max^a^** | **Dose < min^a^** |
| --- | --- | --- |
| ≥ 15 | 0/310 (0%) | 4/310 (1.3%) |
| 12-14 | 19/52 (36.5%) | 0/52 (0%) |
| 6-11 | 0/243 (0%) | 0/243 (0%) |
| 0-5 | 212/1794 (11.8%) | 89/1794 (5.0%) |
| All | 231/2399 (9.6%) | 93/2399 (3.9%) |

^a^ max, maximum dose; min, minimum dose defined by using drug labels or UpToDate .
